# Why is chlorophyll *b* only used in light-harvesting systems?

**DOI:** 10.1007/s10265-018-1052-7

**Published:** 2018-07-10

**Authors:** Atsushi Kume, Tomoko Akitsu, Kenlo Nishida Nasahara

**Affiliations:** 1 Faculty of Agriculture, Kyushu University, 6-10-1 Hakozaki, Higashi-ku, Fukuoka, 812-8581 Japan; 2 Faculty of Life and Environmental Sciences, University of Tsukuba, 1-1-1 Tennodai, Tsukuba, 305-8572 Japan

**Keywords:** Atmospheric optics, Carotenoids, Chlorophyll *c*, Chlorophyll *d*, Spectroradiometer, Terrestrial photosynthesis

## Abstract

Chlorophylls (Chl) are important pigments in plants that are used to absorb photons and release electrons. There are several types of Chls but terrestrial plants only possess two of these: Chls *a* and *b*. The two pigments form light-harvesting Chl *a*/*b*-binding protein complexes (LHC), which absorb most of the light. The peak wavelengths of the absorption spectra of Chls *a* and *b* differ by c. 20 nm, and the ratio between them (the *a*/*b* ratio) is an important determinant of the light absorption efficiency of photosynthesis (i.e., the antenna size). Here, we investigated why Chl *b* is used in LHCs rather than other light-absorbing pigments that can be used for photosynthesis by considering the solar radiation spectrum under field conditions. We found that direct and diffuse solar radiation (PAR_dir_ and PAR_diff_, respectively) have different spectral distributions, showing maximum spectral photon flux densities (SPFD) at c. 680 and 460 nm, respectively, during the daytime. The spectral absorbance spectra of Chls *a* and *b* functioned complementary to each other, and the absorbance peaks of Chl *b* were nested within those of Chl *a*. The absorption peak in the short wavelength region of Chl *b* in the proteinaceous environment occurred at c. 460 nm, making it suitable for absorbing the PAR_diff_, but not suitable for avoiding the high spectral irradiance (SIR) waveband of PAR_dir_. In contrast, Chl *a* effectively avoided the high SPFD and/or high SIR waveband. The absorption spectra of photosynthetic complexes were negatively correlated with SPFD spectra, but LHCs with low *a*/*b* ratios were more positively correlated with SIR spectra. These findings indicate that the spectra of the photosynthetic pigments and constructed photosystems and antenna proteins significantly align with the terrestrial solar spectra to allow the safe and efficient use of solar radiation.

## Introduction

Knowledge of the relationship between the spectrum of incident radiation and the light-harvesting pigments that are found in organisms is crucial for understanding life on Earth and photosynthesis. Various taxa of photosynthetic organisms contain different sets of light-harvesting chlorophylls (Chl). Although several light-harvesting pigments exist, most terrestrial plants use specific Chls (*a* and *b*) and carotenoids to construct pigment-protein complexes (Björn et al. 2009; Esteban et al. 2015; Kiang et al. 2007; Kunugi et al. 2016). By contrast, marine organisms possess a range of Chls (e.g., Chls *a, b, c*_1_, *c*_2_, *c*_3_, *d*, and *f*) and a larger number of accessory pigments, such as carotenoids and/or biliproteins, to allow them to acclimate to the prevailing blue–green light that is found at greater depths (Croce and van Amerongen 2014; Kirk 2011).

**Fig. 1:**
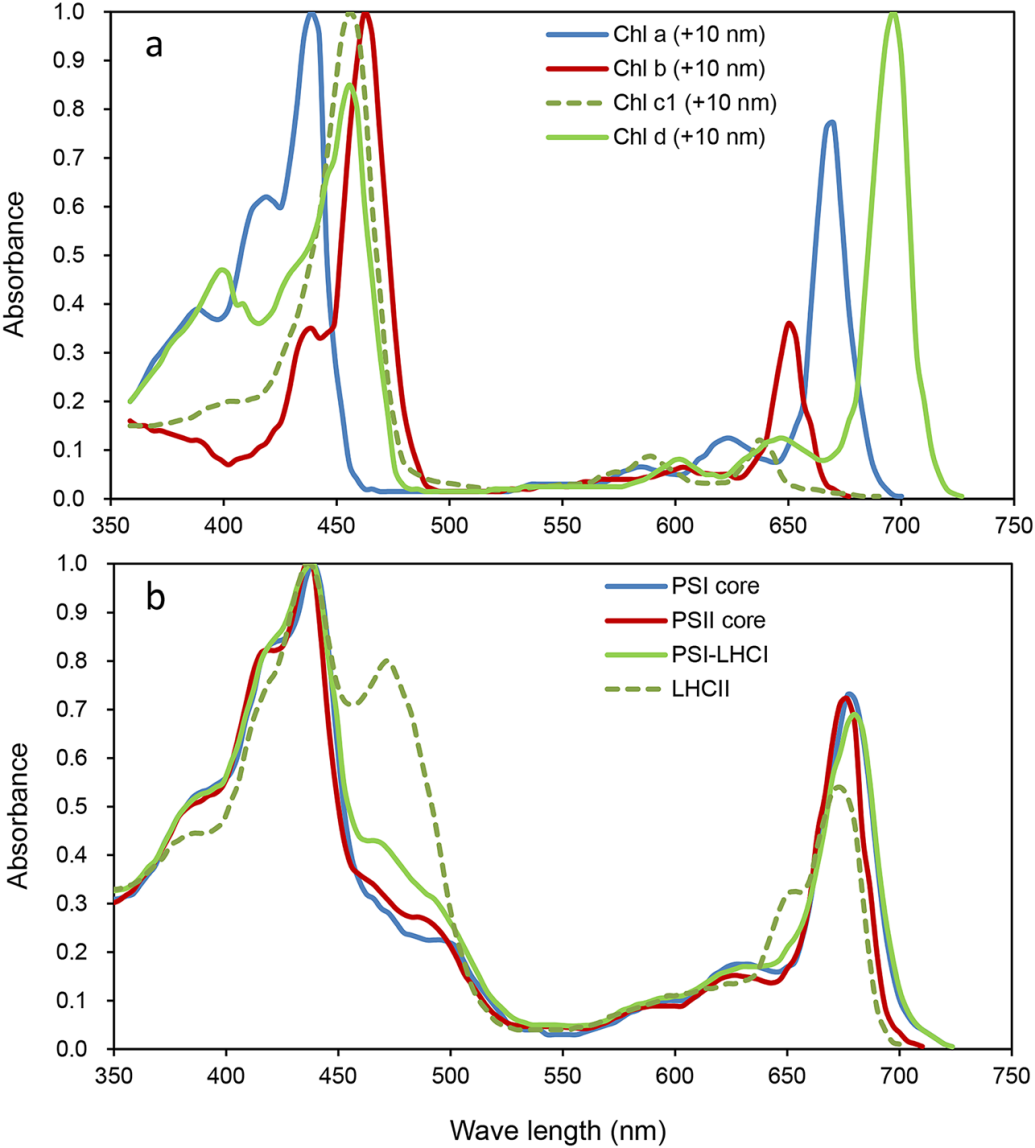
Absorbance spectra of **a** chlorophyll (Chl) *a*, Chl *b*, Chl *c*_1_, and Chl *d*; and **b** photosystem I (PSI) core, PSII core, PSI-light-harvesting complex I (LHCI), and LHCII trimer. Data were taken from Caffarri et al. (2009), Mimuro et al. (2011), Hogewoning et al. (2012), and Bressan et al. (2016). Data were resampled at 3.34-nm intervals and the values less than 0.005 were excluded (see main text for details)

Functional groups (mainly formyl group) at different positions cause different spectral properties, and the absorption maxima of Chls are significantly shifted. Chl *a* is the most abundant of all Chls. Chl *a* is present in the reaction center and light-harvesting complexes of almost all oxygenic photosynthetic organisms including cyanobacteria, algae, and embryophytes. Chl *b* is characteristic for its formyl substitution in C-7 position and is considered as the second most abundant chlorophyll in oxygenic photosynthetic organisms. Kunugi et al. (2016) previously discussed the evolutionary adaptation of incorporating Chl *b* into the light-harvesting system of green plants. It has also been suggested that enhancement of the absorption of green to blue light would be advantageous for photosynthesis in deep water (Larkum 2006; Stomp et al. 2007). Chls *d* and *f* have red-shifted absorption maxima compared with all other Chls and can also utilize infrared light (700–750 nm), which is not absorbed by Chl *a* (Fig. 1a; Chen and Blankenship 2011). Furthermore, Mielke et al. (2011) showed that the energy conversion efficiency of light reaction centers containing Chl *d* is c. 5% better than those containing Chl *a*, with no associated loss in quantum efficiency.

In the terrestrial environment, radiation within the 400–700 nm waveband is defined as photosynthetically active radiation (PAR; McCree 1972), with wavelengths outside this range not being used for photosynthesis (but see Kono et al. 2017). However, Chls do not absorb photons in the PAR waveband evenly (Fig. 1), with only a few percent of absorbance occurring in the green region (500–600 nm), despite the photosynthetic quantum yields being equivalent to those from green and red light (Hogewoning et al. 2012). Chls *a* and *b* function as key components of large protein assemblies known as photosystems. The absorption of light and transfer of energy and electrons mainly occur at photosystem I (PSI), photosystem II (PSII), and light-harvesting Chl *a*/*b*-binding protein complexes (LHC), each of which has significantly different absorbance spectra from their component pigments (Fig. 1a, b). In addition, the leaf tissue structure results in the absorption properties of leaves differing from those of the dissolved pigments (Kume 2017; Moss and Loomis 1952; Vogelmann 1993). However, photon absorption by the chloroplasts is strongly dependent on the absorbance spectra of their constituent pigments (Kume 2017).

The spectrum of incident radiation determines the effectiveness of the absorption spectra of pigments (Kume et al. 2016). Incident global radiation (PAR_glb_) comprises two main components, direct radiation (PAR_dir_), which is sunlight directly transmitted through the atmosphere, and diffuses radiation (PAR_diff_), which is sunlight scattered by the sky and clouds. These components differ greatly in terms of light quantity, directional characteristics, and spectral quality, and depend on the level of cloud cover and various other conditions at a particular site (Akitsu et al. 2015). However, the spectral effects of solar radiation on photosynthesis have been evaluated using averaged spectra, with no consideration for the effects of differences between PAR_dir_ and PAR_diff_.

**Fig. 2:**
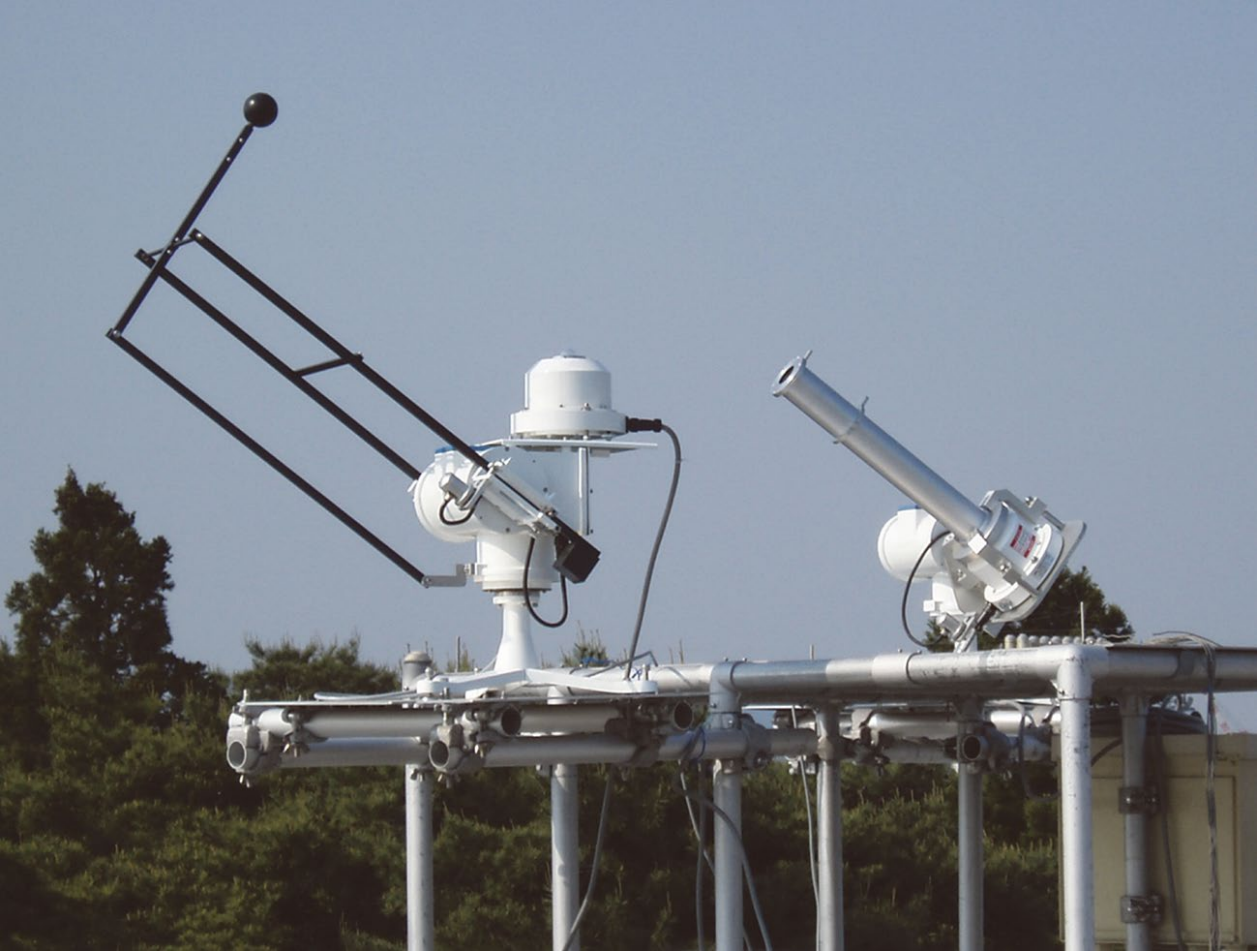
Solar-tracking spectroradiometers set on top of the building of the National Institute for Environmental Studies (NIES) in Tsukuba, central Japan (36.05°N, 140.12°E; 40 m a.s.l.). The spectral range was 350–1050 nm and the spectral interval was 3.3 nm. See Kume et al. (2016) for further details

We previously developed a precise solar-tracking device for detecting direct and diffuse radiation (Fig. 2), and found that the spectral absorbance of the photosystems PSI-LHCI and LHCII decreased linearly with increased spectral irradiance of PAR_dir_ at noon in the high spectral irradiance (SIR) waveband (450–650 nm) and the spectral absorbance of Chl *a* was strongly negatively correlated with the SIR (W m^−2^ nm^−1^) of PAR_glb_ at noon (Kume et al. 2016). These findings suggested that terrestrial green plants have evolved to reduce excess energy absorption rather than to absorb PAR photons efficiently, and that the absorption spectra of Chls *a* and *b* may interact strongly with the spectral differences between PAR_dir_ and PAR_diff_ (Kume 2017; Kume et al. 2016). However, the adaptive significance of Chl *b* remains unclear.

In this study, we tested the hypothesis that the solar spectra of PAR_dir_ and PAR_diff_ have been the evolutionary pressure behind terrestrial plants only using Chls *a* and *b* among the range of Chls that are available, and that the absorption spectra of photosystems are adapted for life in a terrestrial environment. To do this, we analyzed the relationship between the incident PAR_dir_ and PAR_diff_ spectra and the absorption spectra of the light-harvesting pigments (Chls *a, b, c*_1_, and *d*) and photosynthetic complexes (PSI core, PSII core, PSI-LHCI, and LHCII trimer).

## Materials and methods

Shortwave radiation (SW) data obtained by Kume et al. (2016) on a sunny day (day of year [DOY] 195) and a cloudy day (DOY 175) in 2011 were used in this study. Measurements were made at 1-min intervals during daylight from the top of the National Institute for Environmental Studies (NIES) building in central Japan (36.05°N, 140.12°E), from where we could conveniently define direct radiation as that which occurred within a 5° angle of the direction of the sun and diffuse radiation as that which did not originate from the direction of the sun. Global radiation is defined as the sum of direct and diffuse radiation.

To investigate how the spectral absorption features of different Chls and photosystems interact with the different spectra of PAR_dir_ and PAR_diff_, we calculated the correlation coefficient between the absorbance spectrum of each pigment (Fig. 1; the wavelength range from 400 to 720 nm at 3.34-nm intervals, values less than 0.005 were excluded) and the spectral irradiance or spectral photon density for PAR_dir_ and PAR_diff_ at 1-min intervals.

The absorbance spectra of Chls *a, b, c*_1_, and *d* were taken from Mimuro et al. (2011), while those of the PSI core and PSI-LHCI were taken from Bressan et al. (2016), that of the PSII core was taken from Caffarri et al. (2009), and that of the LHCII trimer was taken from Hogewoning et al. (2012). The Chl *a*/*b* ratio is 28.83 for the PSI core (Bressan et al. 2016), 7.06 for PSI-LHCI (Bressan et al. 2016), and 1.3 for the LHCII trimer (Hogewoning et al. 2012). The PSI core and PSII core almost completely lacks Chl *b* (Caffarri et al. 2009; Esteban et al. 2015).

It was noted that the spectral profiles of Chl absorbance measured in vitro differ slightly from those obtained in the protein environment, limiting any discussion that is based on a single absorption spectrum of a pigment dissolved in organic solvent. French et al. (1972) showed that the absorption spectra of Chl *a* in the photosystems have several peaks in the 640–690 nm region. However, Chl absorbance spectra that are measured in vitro are always significantly blue-shifted compared with those measured *in vivo*, with Cinque et al. (2000) reporting that the proteinaceous environment induces a red-shift of c. 10 nm in the main absorption peaks compared with the absorption spectra of pigments dissolved in organic solvent. Cinque et al. (2000) have demonstrated that, in addition to the expected red-shift of the optical transition, the overall shapes and spectral forms of both Chls within the proteinaceous environment are rather similar to their corresponding absorption spectra in an organic solvent. Therefore, in order to reflect the proteinaceous environment, we shifted the spectral data of the Chls by + 10 nm (Fig. 1a).

## Results

### Diurnal changes in the solar spectra

**Fig. 3:**
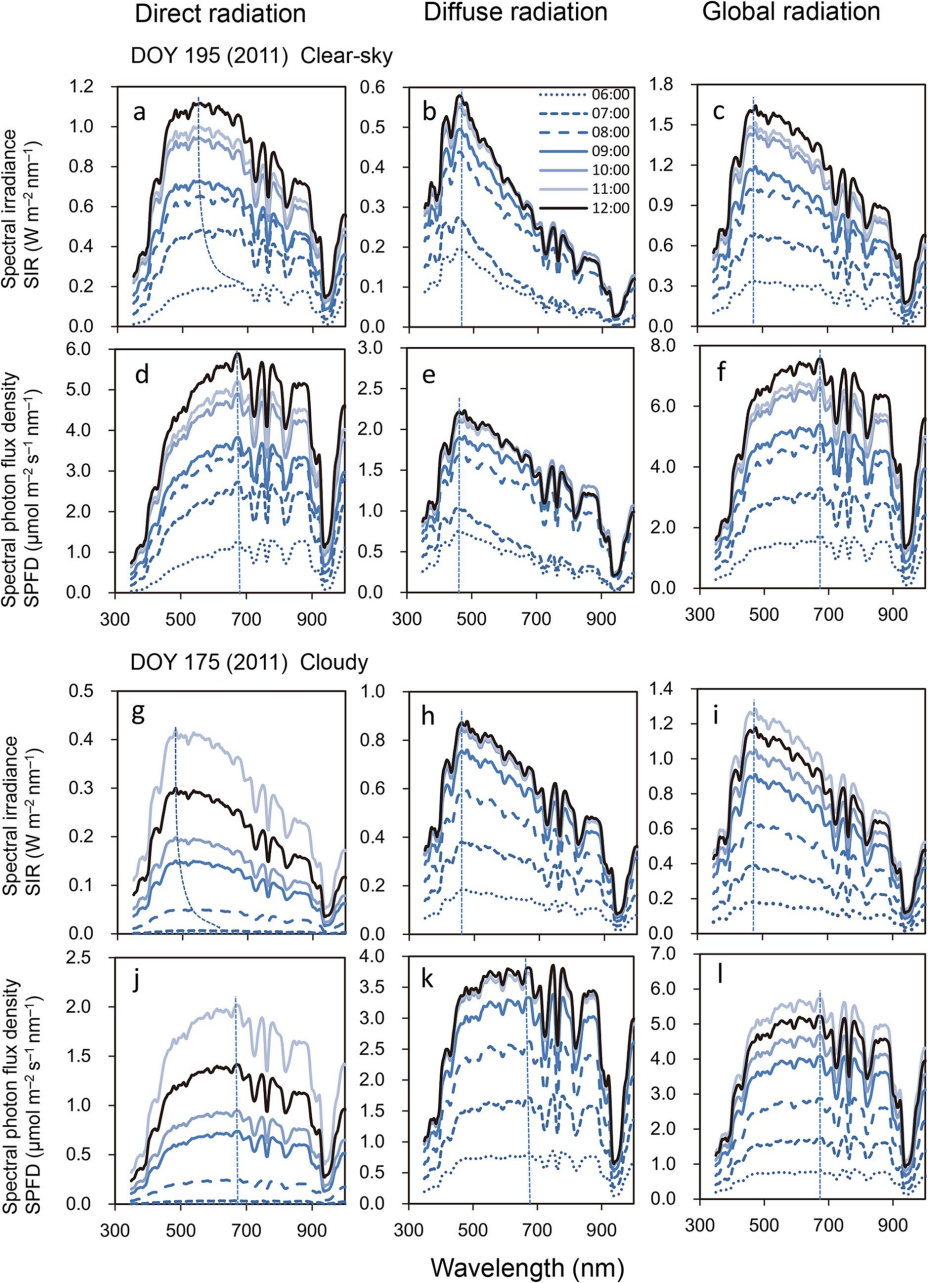
Spectral irradiance (SIR) and spectral photon flux density (SPFD) of shortwave radiation (SW) on a clear-sky morning (**a**–**f**) and cloudy morning (**g**–**l**). Direct (**a, d, g, j**) diffuse (**b, e, h, k**) and global (**c, f, i, l**) radiation. Measurements were made on day of year 195 (clear-sky) and 175 (cloudy) in 2011 and were averaged over each hour. Dashed lines indicate peak wavelengths (λ_max_). ${\text{PAR}}_{{{\text{dir}}}}^{{\text{E}}}$ (**a, g**), ${\text{PAR}}_{{{\text{diff}}}}^{{\text{E}}}$ (**b, h**), ${\text{PAR}}_{{{\text{glb}}}}^{{\text{E}}}$ (**c, i**), ${\text{PAR}}_{{{\text{dir}}}}^{P}$ (**d, j**), ${\text{PAR}}_{{{\text{diff}}}}^{{\text{P}}}$ (**e, k**), ${\text{PAR}}_{{\operatorname{glb} }}^{{\text{P}}}$ (**f, l**)

**Fig. 4:**
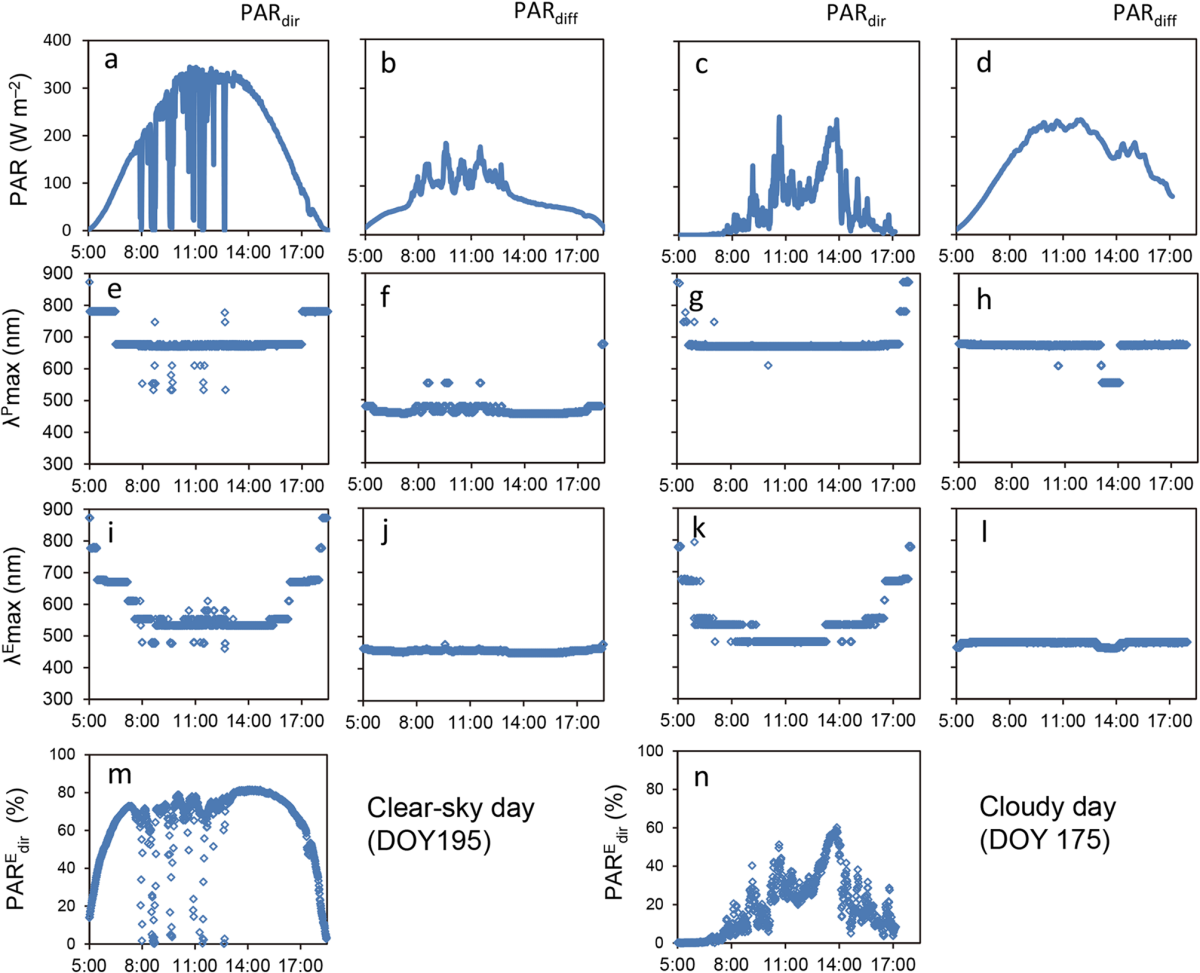
Direct and diffuse photosynthetically active radiation (PAR) on a clear-sky day (left panels) and a cloudy day (right panels) at Tsukuba. Measurements were made every minute on day of year 195 and 175 in 2011. **a**–**d** PAR intensity (W m^− 2^), **e**–**h** peak wavelength of spectral photon density ($\lambda _{{\hbox{max} }}^{{\text{P}}}$), **i**–**l** peak wavelength of spectral irradiance ($\lambda _{{\hbox{max} }}^{{\text{E}}}$), and **m**–**n** percentage of direct radiation of PAR (${\text{PAR}}_{{{\text{dir}}}}^{{\text{E}}}$) per global radiation of PAR (${\text{PAR}}_{{{\text{glb}}}}^{{\text{E}}}$)

Typical SW spectra on a sunny day are shown in Figs. 3 and 4. In the morning, the SIR spectrum of PAR_dir_ (${\text{PAR}}_{{{\text{dir}}}}^{{\text{E}}}$ ) displayed a broad peak, with a maximum ($\lambda _{{\hbox{max} }}^{{\text{E}}}$) at around 700 nm (Figs. 3a, 4i). As the day progressed (i.e., as the solar zenith angle decreased), $\lambda _{{\hbox{max} }}^{{\text{E}}}$ shifted toward shorter wavelengths to reach 540 nm at noon (solar zenith angle = 14.5°), at which time the SIR exceeded 1.0 Wm^− 2^ nm^− 1^ (Fig. 3a). Overall, ${\text{PAR}}_{{{\text{dir}}}}^{{\text{E}}}$ contributed c. 80% of the global PAR energy (Fig. 4m). By contrast, the $\lambda _{{\hbox{max} }}^{{\text{E}}}$ of ${\text{PAR}}_{{{\text{diff}}}}^{{\text{E}}}$ exhibited no such diurnal changes, remaining almost constant at 455 nm (Figs. 3b, 4j). Similarly, while the SPFD spectrum of PAR_dir_ (${\text{PAR}}_{{{\text{dir}}}}^{{\text{P}}}$) displayed a broad fixed peak ($\lambda _{{\hbox{max} }}^{{\text{P}}}$) at 680 nm (Figs. 3d, 4e) and the SPFD exceeded 5.0 µmol m^− 2^ s^− 1^ nm^− 1^ (Fig. 3d), the $\lambda _{{\hbox{max} }}^{{\text{P}}}$ of ${\text{PAR}}_{{{\text{diff}}}}^{{\text{P}}}$ remained at around 480 nm throughout the day (Figs. 3e, 4f). The SIR spectra of global radiation (${\text{PAR}}_{{{\text{glb}}}}^{{\text{E}}}$) exhibited similar profile to those of ${\text{PAR}}_{{{\text{diff}}}}^{{\text{E}}}$ (Fig. 3b, c), but the SPFD spectra of global radiation (${\text{PAR}}_{{{\text{glb}}}}^{{\text{P}}}$) were similar to ${\text{PAR}}_{{{\text{dir}}}}^{{\text{P}}}$ (Fig. 3d, f).

Diurnal changes in ${\text{PAR}}_{{{\text{dir}}}}^{{\text{E}}}$ on the cloudy day were similar to those observed on the sunny day (Fig. 3a, g), but $\lambda _{{\hbox{max} }}^{{\text{E}}}$ was slightly shorter (Fig. 4i, k) and the ${\text{PAR}}_{{{\text{dir}}}}^{{\text{E}}}$ energy contribution was also quite small, being negligible during the morning and less than 50% during the daytime (Fig. 4n). This indicates that most of the ${\text{PAR}}_{{{\text{dir}}}}^{{\text{E}}}$ that is detected on cloudy days may comprise ${\text{PAR}}_{{{\text{diff}}}}^{{\text{E}}}$ coming from the direction of the sun. Both the ${\text{PAR}}_{{{\text{diff}}}}^{{\text{P}}}$ and ${\text{PAR}}_{{\operatorname{glb} }}^{{\text{P}}}$ spectra were relatively flat from c. 500 to 700 nm (Fig, 3 k, l) and all PAR^P^ spectra peaked locally at around 670 nm (Figs, 3j–l, 4g, h).

Together, these findings indicate that absorption in the 650–700 nm bandwidth is suitable for ${\text{PAR}}_{{{\text{dir}}}}^{{\text{P}}}$ (Figs. 3d, 3j, 4e, 4g) and ${\text{PAR}}_{{\operatorname{glb} }}^{{\text{P}}}$ (Fig. 3f, l), while absorption in the 450–500 nm bandwidth is suitable for ${\text{PAR}}_{{{\text{dir}}}}^{{\text{P}}}$ under a clear sky (Fig. 3e). However, the 500–600 nm bandwidth is also important for ${\text{PAR}}_{{{\text{dir}}}}^{{\text{E}}}$ to avoid strong SIR at noon (Figs. 3a, 4i).

### Relationship between solar radiation and pigment absorption spectra

To examine the interactions between the spectral absorption characteristics of the pigments and photosystems and the different PAR_dir_ and PAR_diff_ spectra, we calculated correlation coefficients between the spectral absorbance of each pigment or photosystem and the spectral photon density (*r*_p_) and irradiance (*r*_e_) of PAR_dir_ and PAR_diff_ at 1-min intervals based on the SPFD (PAR^P^; µmol m^− 2^ s^− 1^ nm^− 1^) and the SIR (PAR^E^; W m^− 2^ nm^− 1^), respectively. A positive correlation coefficient indicated that spectral absorption tended to increase with spectral quantum density or irradiance or, in this case, that the pigment tended to absorb more radiation of wavelengths with a higher quantum density or irradiance.

**Fig. 5:**
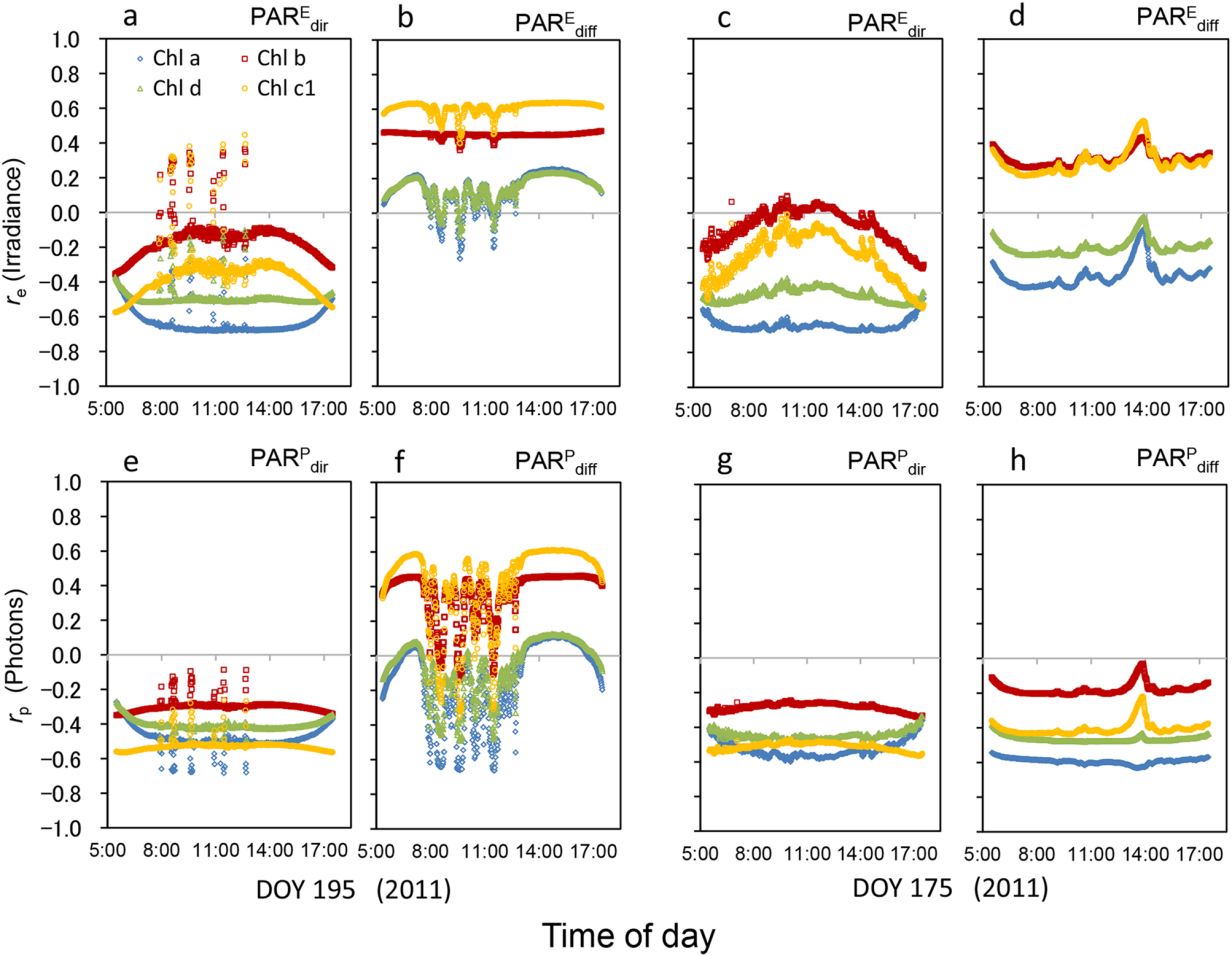
Correlation coefficients between the spectral absorbance of chlorophyll (Chl) *a*, Chl *b*, Chl *c*_1_, and Chl *d* and the spectral irradiance (SIR; Wm^− 2^ nm^− 1^) (*r*_e_; **a**–**d**) and spectral photon flux density (SPFD; µmol m^− 2^ s^− 1^ nm^− 1^) (*r*_p_; **e**–**h**) for direct and diffuse photosynthetically active radiation (PAR_dir_ and PAR_diff_, respectively). Measurements were made at 3.34-nm wavelength intervals every 1 min on a clear-sky day (day of year [DOY] 195; **a, b, e, f**) and a cloudy day (DOY 175; **c, d, g, h**) in 2011 at Tsukuba

In general, Chl *a* showed a strong negative correlation with irradiance regardless of the sky condition or PAR class, with *r*_e_ for ${\text{PAR}}_{{{\text{dir}}}}^{{\text{E}}}$ under a clear sky being c. −0.68 (Fig. 5a), indicating that Chl *a* absorbed less of the bands with a larger spectral irradiance of PAR_dir_. By contrast, Chl *b* was consistently positively associated with the irradiance of PAR_diff_, with *r*_e_ and *r*_p_ for PAR_diff_ under a clear sky being c. 0.45 (Fig. 5b, f) and this tendency being maintained even on the cloudy day (Fig. 5c, d, g, h). This indicates that Chl *b* absorbed photons of PAR_diff_ more efficiently than Chl *a*. The *r*_e_ values of Chl *c*_1_ were similar to those of Chl *b* but the *r*_p_ values of Chl *c*_1_ were lower than those of Chl *b* with the exception of PAR^P^_diff_ (Fig. 5f). The *r*_p_ and *r*_e_ values of Chl *d* consistently fell between those of Chl *a* and Chl *b*, and were relatively steady regardless of the PAR class (Fig. 5).

**Fig. 6:**
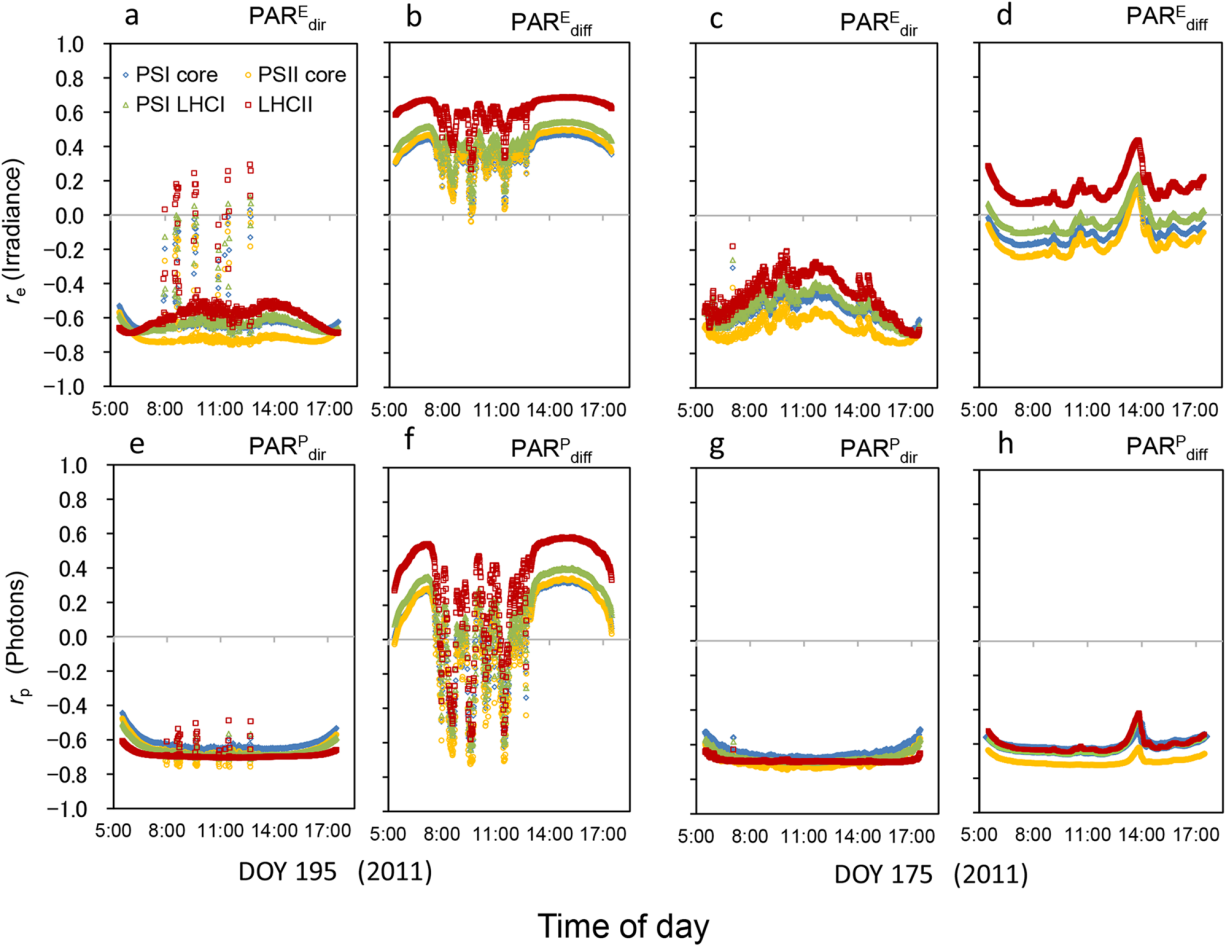
Correlation coefficients between the spectral absorbance of each photosystem (photosystem I [PSI] core, PSII core, PSI-light-harvesting complex I (LHCI), and LHCII trimer) and the spectral irradiance (SIR; Wm^− 2^ nm^− 1^) (*r*_e_; **a**–**d**) and spectral photon flux density (SPFD; µmol m^− 2^ s^–1^ nm^− 1^) (*r*_p_; **e**–**h**) for direct and diffuse photosynthetically active radiation (PAR_dir_ and PAR_diff_, respectively). Measurements were made at 3.34-nm wavelength intervals every 1 min on a clear-sky day (day of year [DOY] 195; **a, b, e, f**) and a cloudy day (DOY 175; **c, d, g, h**) in 2011 at Tsukuba

The PSI and PSII cores showed strong negative correlations with PAR_dir_ regardless of the sky condition and radiation unit (Fig. 6a, c, e, g), with *r*_e_ and *r*_p_ for PAR_dir_ under a clear sky being c. − 0.7 (Fig. 6a, e). Furthermore, these photosystems showed more negative *r*_e_ and *r*_p_ values than Chl *a* in the early morning and late afternoon (Figs. 5, 6). By contrast, LHCII trimer showed more positive correlations than the other photosystems, particularly for ${\text{PAR}}_{{{\text{diff}}}}^{{\text{E}}}$ (Fig. 6b, d).

### Sky and pigment absorption spectra

**Fig. 7:**
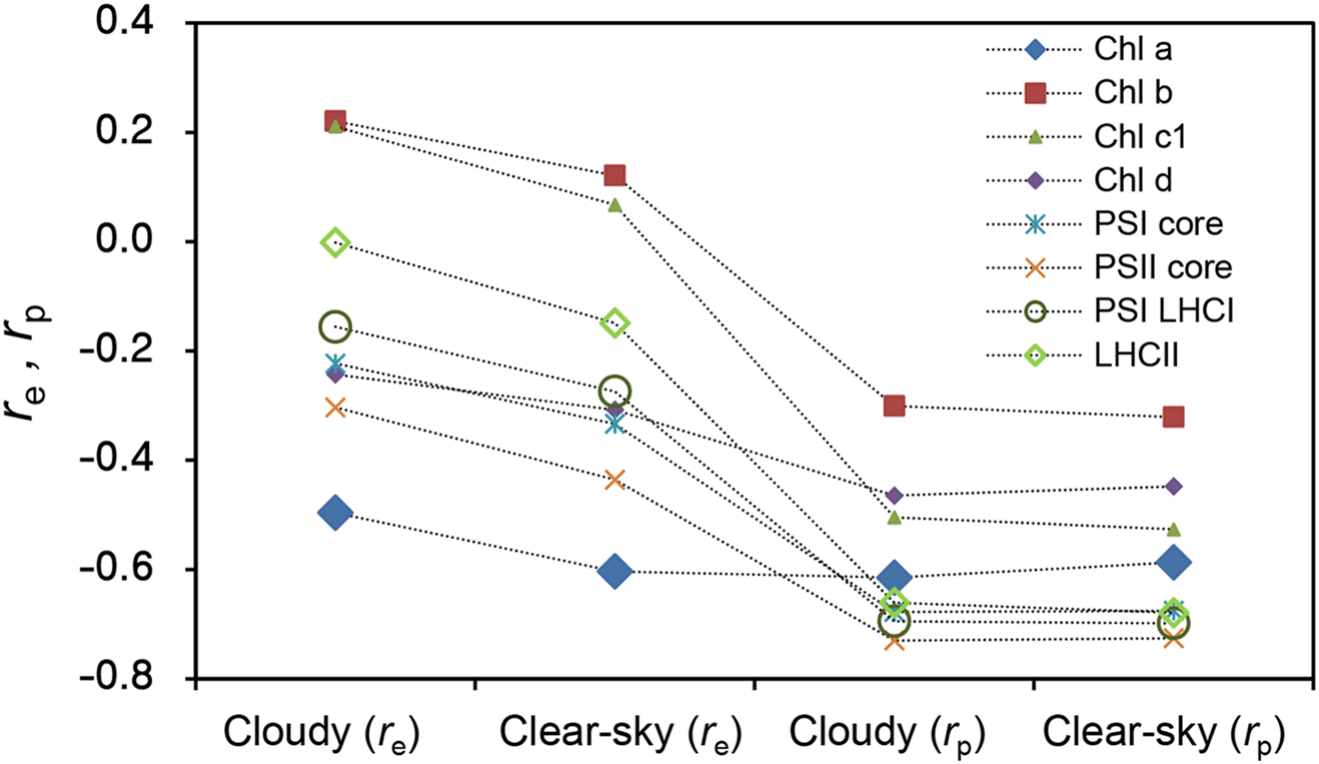
Correlation coefficients between the spectral absorbance of chlorophylls (Chls *a, b, c*_1_, and *d*) and photosystems (photosystem I [PSI] core, PSII core, PSI-light-harvesting complex I (LHCI), and LHCII trimer) and the spectral irradiance (SIR; Wm^− 2^ nm^− 1^) (*r*_e_) and spectral photon flux density (SPFD; µmol m^− 2^ s^–1^ nm^− 1^) (*r*_p_) for global solar radiation on a clear-sky day (day of year = 195) and cloudy day (day of year = 175) in 2011 at noon (36.05° N, 140.12° E). Measurements were conducted at 1-min intervals averaged over 1 h (11:30 am–12:30 pm)

To examine the interactions between the spectral absorption characteristics of the pigments and photosystems and the different sky spectra, the correlation coefficients between the spectral absorbance of each pigment or photosystem and the PAR_glb_ spectra at noon were compared (Fig. 7). Among the photosynthetic pigments, Chl *a* consistently showed the most negative correlation coefficient (*r*_e_, *r*_p_), whereas Chl *b* consistently showed the most positive correlation coefficient under any sky condition. The correlation coefficients of Chl *c*_1_ and *d* were located between them. These results suggest that Chls *a* and *b* show the most contrasting absorption characteristics in the terrestrial radiation environment and that they are functionally differentiated. PSI and PSII cores and PSI-LHCI and LHCII showed the most negative *r*_p_ compared with any other single Chl species in the SPFD, while they showed more positive *r*_e_ in the SIR and the values consistently fell between those of Chl *a* and Chl *b* (Fig. 7).

## Discussion

### Why were Chls *a* and *b* selected for in the terrestrial environment?

The absorption spectrum of Chl *a* exhibited a consistent tendency to avoid PAR_dir_ and it produces consistent negative *r*_e_ and *r*_p_ of PAR_glb_, while that of Chl *b* tended to absorb PAR_diff_, suggesting that Chl *a* can effectively avoid strong, direct solar radiation and Chl *b* can efficiently use diffused solar radiation. These differences are caused by slight shifts in the position and band width of absorption peaks in the blue and red regions (Fig. 1a), with the high absorption efficiency of Chl *b* for PAR_glb_ in the terrestrial environment being related to it having a higher Soret absorption band than Chl *a* and the longest Soret wavelength among the Chl pigments (c. 452 nm in diethyl ether; Mimuro et al. 2011). Such functional differences between the photosynthetic pigments appears to be quite adaptive for life in the terrestrial radiation environment and suggest that green algal progenitors were selected from the many other photosynthetic organisms living in an aquatic environment with different photosynthetic pigments (Björn et al. 2009; Kunugi et al. 2016).

Kunugi et al. (2016) suggested that the elimination of Chl *b* from PSI core antennae contributed greatly to the evolution of terrestrial green plants. To extend this concept, we analyzed the absorption spectra of Chls *c*_1_ and *d*. Chl *c*_1_ is a common form of Chl *c*. It is widely distributed among the secondary endosymbionts derived from red algae and is suitable for the light conditions of their marine habitats (Garrido et al. 1995). Chl *c* functions together with Chl *a* and carotenoids as light-harvesting pigments. Chl *a* shows only weak absorbance between 450 and 650 nm, while Chl *b* or *c* show increased absorbance within this range at both the long- and short-wavelength ends (Kirk 2011). The *r*_e_ for PAR_dir_ and PAR_diff_ of Chl *c*_1_ were similar to those of Chl *b*, but its *r*_p_ values, especially on the cloudy day, were lower than those of Chl *b* (Fig. 5g, h). The *r*_p_ and *r*_e_ for PAR_glb_ of Chl *c*_1_ lied between those of Chls *a* and *b* (Fig. 7). The peak absorbance of Chl *c*_1_ at the long-wavelength end is significantly smaller than that of Chl *b* (Fig. 1a); thus, the absorption of photons by Chl *c*_1_ in the long-wavelength region becomes much lower than that by Chl *b*. As a result, Chl *c*_1_ does not surpass Chl *b* as a light-harvesting pigment in the terrestrial environment, where longer wavelength photons are abundant.

Chl *d* is only found in a few cyanobacteria inhabiting aquatic environments (Kashiyama et al. 2008) and constitutes part of the light reaction center complex rather than merely occurring as an accessory pigment (Mielke et al. 2011). Interestingly, the *r*_p_ and *r*_e_ values of Chl *d* consistently lay in between those of Chls *a* and *b* and remained relatively constant regardless of the PAR class (Figs. 5, 7). Thus, it appears that aquatic Chl *d* would not be well suited to the terrestrial, direct-diffuse radiation environment, as its absorption characteristics would be unsatisfactory for avoiding or gathering solar radiation.

It is noted that we used + 10 nm shifted spectral data of Chls in the current study to reflect the proteinaceous environment (Fig. 1a). Interestingly, however, this corrected dataset had a similar but rather weak correlation with the spectral solar radiation in comparison with the previous research (Kume et al. 2016).

### What is the advantage of forming pigment-protein complexes?

The spectrum of incident radiation determines the effectiveness of the absorption spectra of pigments, but Chl biosynthesis and its regulation in the embryophytes depend on: plant species, developmental stage and environmental factors, such as light conditions, temperature, and the composition of ambient atmosphere. Thus, chlorophyll formation may be regulated on various levels. It is well established that the Chl *a*/*b* ratio increases in unshaded conditions (i.e., when exposure to PAR_dir_ is high) and decreases in shadier environments (i.e., when relative PAR_diff_ is elevated). This phenomenon occurs across all magnitudes of scale, from intra-chloroplast (Anderson et al. 1988) through to the leaves (Terashima 1989) and the whole plant (Bordman 1977). Furthermore, Kume and Ino (1993) observed clear seasonal changes in the Chl *a*/*b* ratio in the leaves of evergreen, broadleaved shrubs. Chls and carotenoids in the plant thylakoid membranes form pigment-protein complexes. Chl *b* occurs exclusively in LHCs, which function as peripheral antennae (Kunugi et al. 2016). In green plants, the antenna size of PSII is determined by the amount of LHCII (Jansson 1994; Tanaka and Tanaka 2011) and levels of LHCII are highly correlated with the accumulation of Chl *b* (Bailey et al. 2001; Jia et al. 2016), which is synthesized from Chl *a* by chlorophyllide *a* oxygenase (Tanaka and Tanaka 2011; Yamasato et al. 2005). When plants grow under low light intensities, Chl *b* synthesis is enhanced and the antenna size increases (Bailey et al. 2001). Since LHCII is the major light-harvesting complex of plants and the most abundant membrane protein, the absorption spectrum of the LHCII trimer may represent the average chloroplast absorption spectrum (Kume 2017). The absorption spectrum of LHCII is significantly different from a single Chl molecule or the core photosystems, particularly with regard to the secondary absorption peak that occurs at 472 nm with a shoulder at 653 nm (Fig. 1b).

**Fig. 8:**
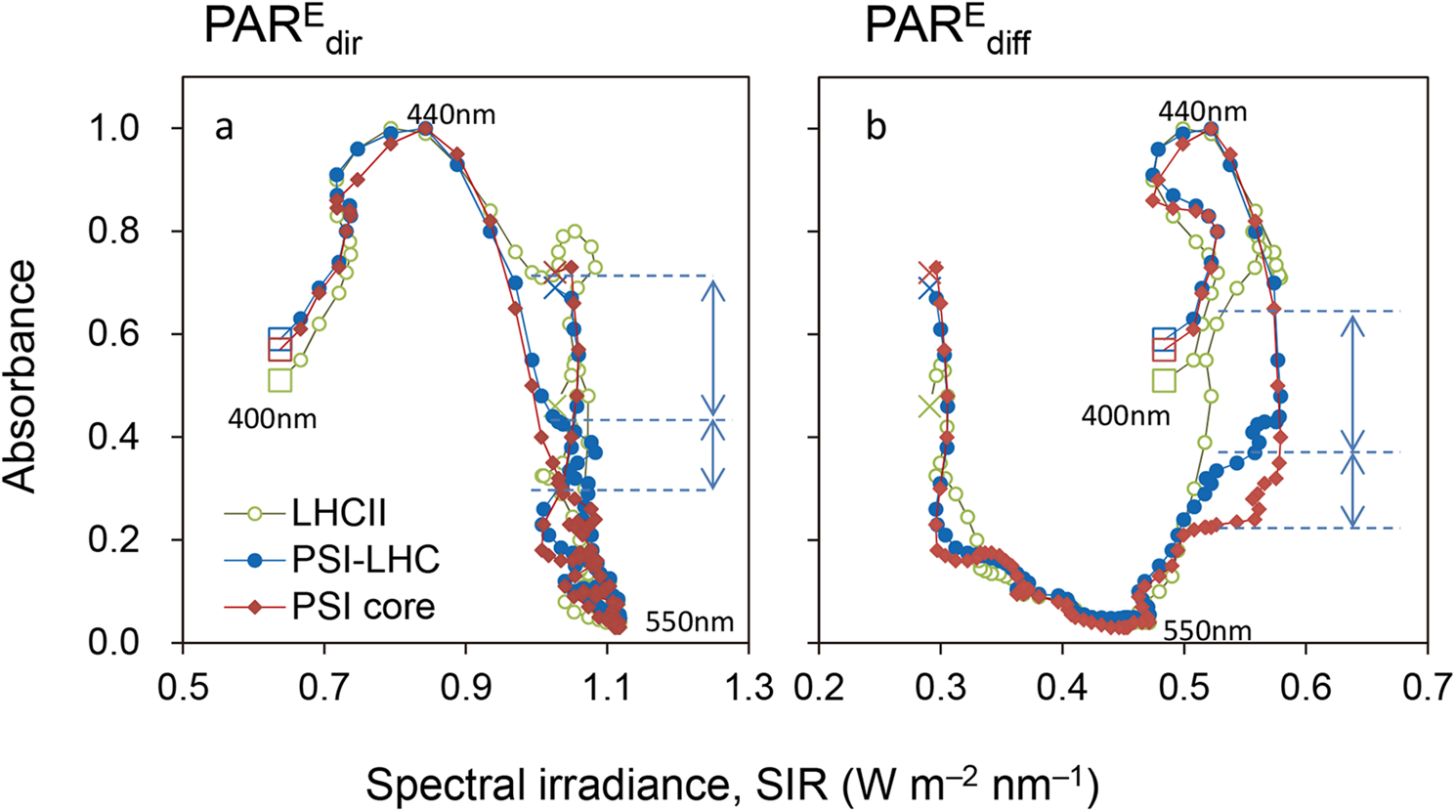
Relationships between the spectral absorbance of the light-harvesting complex II (LHCII) trimer, photosystem I (PSI)-LHCI, and the PSI core and the spectral irradiance of (**a**) direct solar photosynthetically active radiation (PAR) and (**b**) diffuse solar PAR at noon. Spectral absorbance is plotted on the *y*-axis and spectral irradiance on the *x*-axis at 3.35-nm intervals in the 400–680-nm bandwidth. Points with consecutive wavelengths are connected with a line. The points with the shortest (400 nm) and longest (680 nm) wavelengths are indicated by squares and crosses, respectively. The horizontal dashed lines indicate the absorbance of each photosystem at 570 nm and the vertical arrows indicate the difference between these, which is mainly caused by the amount of Chl *b* contained in each photosystem or antenna

Preventing excess energy absorption in photosystems is an essential survival strategy in terrestrial environments, where the atmospheric CO_2_ concentration is too low to utilize incident solar radiation safely for photosynthesis and the photon flux density can fluctuate by several orders of magnitude (Kume 2017; Ruban 2015). Kume et al. (2016) found that the spectral absorbance of Chl *a* is strongly negatively correlated with the spectral irradiance of PAR_glb_ at noon and Kunugi et al. (2016) showed that the exclusion of Chl *b* from the core antennae is crucial for promoting high-light resistance. In the present study, we found that PSI and PSII cores, which do not include Chl *b*, showed strong negative *r*_e_ and *r*_p_ values under PAR_dir_, and that these values tended to be more negative than those for Chl *a*. However, the addition of LHCI, which includes Chl *b*, to PSI to form PSI-LHCI led to an increase in *r*_e_, while the LHCII trimer, which has the lowest *a*/*b* ratio, showed the highest *r*_e_ values. These differences were mainly caused by differences in absorbance in the vicinity of the 470-nm waveband (Figs. 1b, 8). The increase in Chl *b* in LHCs raises the absorbance at the high SIR waveband rather than that at the high SPFD waveband.

The spectra of photosystems and LHCs are consistently adjusted to avoid the high SPFD waveband (Fig. 8a). However, the spectra of photosystems and LHCs are different because of different Chl *b* contents and have complementary functional relationships. Compared with PSI and PSII cores, LHCII shows higher absorbance on the short-wavelength end and a relatively lower absorbance on the long-wavelength end (Fig. 1b). The peak spectral absorbance in the high SIR waveband (< 520 nm) is high and that in the high SPFD waveband (> 670 nm) is low. Therefore, although *r*_p_ values of LHCII are only slightly different from those of PSI and PSII cores, the total spectral absorbance increases with the combination of cores and LHCs.

The *r*_p_ values of all pigment-protein complexes showed strong negative correlation with the exception of ${\text{PAR}}_{{{\text{diff}}}}^{{\text{P}}}$. This is the results of absorption by carotenoids in the complexes. Among carotenoids, β-carotene is almost exclusively located in PSI and PSII cores, and lutein and other carotenoids are located in LHCs (Esteban et al. 2016). These carotenoids absorb high SIR photons (400–520 nm) without attenuation in high SPFD photons (550–700 nm), and reduce the absorption of high SIR photons by Chls (Kume et al. 2016). Kume (2017) previously discussed the filtering effects of accessory pigments and has defined the surplus energy (Es) as the part of energy potentially exchanged as heat in the absorbed photon energy. The absorption spectra of carotenoids are quite effective for eliminating photons that produce high Es. Since carotenoids are functioning both in light capture and photoprotection, further studies are required to understand the functional differentiation of carotenoids in pigment-protein complexes.

Notably, LHCII is the peripheral antenna for PSII and can associate with PSI depending on light conditions (e.g., Benson et al. 2015; Grieco et al. 2015). The LHCI complexes mediate energetic interaction between “extra” LHCII and PSI core in the intact membrane (Benson et al. 2015; Grieco et al. 2015). Plants have a much higher ability to dissipate the light energy absorbed by the LHCII antenna as heat. This could be the one of the major reasons to protect the core antenna from strong solar radiation.

### Why do plants absorb less green light?

Since light-use efficiency is an important component of biomass production, several leaf photosynthesis models have been proposed that consider the light absorption profile based on the optimal use of PAR photons in the terrestrial environment. Most discussions around this have focused on the efficient use of incident PAR photons in photosynthesis. However, the relationships between the spectral characteristics of incident radiation from the sun and the energy balance of chloroplasts and pigment characteristics, and the ways in which these affect leaf physiological conditions are also crucially important (Kume 2017).

The waveband of the green region of the spectrum (500–570 nm) is identical to that of strong, directional solar irradiance at midday under a clear sky (Figs. 3a, 4i). Kume et al. (2016) showed that the spectral absorbances of photosystems PSI-LHCI and LHCII and intact leaves decrease linearly with the increased spectral irradiance of $PAR_{{{\text{dir}}}}^{{\text{E}}}$ at noon in the high spectral irradiance waveband (450–650 nm). In the present study, the PSI and PSII cores, which do not contain Chl *b*, showed the lowest absorbance in the vicinity of the 460-nm waveband (Figs. 1a, 8), which contrasts with marine photosynthetic organisms that are adapted to enhance absorption efficiency in the 450–650 nm wavelength range. Consequently, changes in the light-harvesting system may have contributed greatly to the evolution of terrestrial green plants, which are fine-tuned to reduce excess energy absorption rather than to absorb PAR photons efficiently. As Ruban (2015) emphasized, the photosynthetic antenna was “reinvented” a number of times in the course of evolution and hence originates from multiple ancestors. The photochemical reaction center and core antennae of terrestrial plants only include Chl *a*, which has low solar radiation absorptivity, with the peripheral antenna complex containing Chl *b* and carotenoids being arranged around this. The energy state of LHCII is precisely regulated and balanced by various photochemical mechanisms (Galka et al. 2012; Ruban 2015), resulting in plants being protected from high PAR while achieving high light absorption efficiency.

It is well known that light is the most limiting resource for plant growth and that competition between plants affects their various responses to environmental changes (Anten 2005; Givnish 1988; van Loon et al. 2014). Thus, the efficient use of PAR under cloudy or shaded conditions may be important. On sunny days, PAR_dir_ contributes more than 80% to the incident global PAR energy (Fig. 4m), but this decreases to less than 50% on cloudy days and almost 0% on cloudy mornings (Fig. 4n). By contrast, PAR_diff_ remains relatively stable in terms of the amount of incident energy and λ_max_. These spectral differences between PAR_dir_ and PAR_diff_ ensure that diffuse solar radiation, which has much less tendency to cause canopy photosynthetic saturation, is used more effectively by plant canopies than direct solar radiation. Thus, our findings suggest that the absorption spectrum of LHCII enables the efficient use of PAR_diff_ and cloudy-day radiation, and that diffuse and direct radiation trigger different responses in canopy photosynthesis. The changeability of the LHC antenna size, which is reflected in changes in spectral absorption, has a major effect on the distribution of plants as it allows flexibility in PAR use efficiency and avoidance of the strong heat produced by PAR_dir_ (e.g., Murchie and Horton 1997). Thus, leaves that are exposed to sun and shade may be regarded as PAR_dir_ and PAR_diff_ adapted, respectively.

Notably, the effects of spectral differences between PAR_dir_ and PAR_diff_ are negligible for whole-leaf absorption properties. Kume (2017) has demonstrated that the absorption spectra of the intact leaves of terrestrial plants function as a gray body. The photon absorption of the whole leaf is efficiently regulated by photosynthetic pigments through a combination of pigment density distribution and leaf anatomical structures. The spectral characteristics of absorbers are important factors for the energy regulation of chloroplasts and smaller-scale energy processes.

## Conclusion

Our findings indicate that the absorbance spectra of photosynthetic pigments and the photosystems and antenna proteins they construct significantly align with the spectra of PAR_dir_ and PAR_diff_ to enable the safe and efficient use of solar radiation on land. Chl *b* tends to absorb scattered solar radiation complementary to Chl *a* and so its incorporation into the peripheral antennae increases the absorption capacity of plants for this type of radiation. By contrast, Chl *b* is excluded from the core antennae to avoid the absorption of strong, direct solar radiation. Thus, it appears that the effect of the absorption and scattering of solar radiation in the atmosphere on the spectral absorption of photosynthetic organisms was a primary driver of the selection of photosynthetic pigments and the evolution of photosystems. This research adds to the growing body of evidence that suggests that terrestrial green plants are fine-tuned to the spectral and temporal dynamics of incident solar radiation. However, further field observations and analyses are required to better understand the spectral adaptation of terrestrial organisms.
