# Correction to: Why is chlorophyll *b* only used in light-harvesting systems?

**DOI:** 10.1007/s10265-019-01104-y

**Published:** 2019-04-07

**Authors:** Atsushi Kume, Tomoko Akitsu, Kenlo Nishida Nasahara

**Affiliations:** 1 Faculty of Agriculture, Kyushu University, 6-10-1 Hakozaki, Higashi-ku, Fukuoka, 812-8581 Japan; 2 Faculty of Life and Environmental Sciences, University of Tsukuba, 1-1-1 Tennodai, Tsukuba, 305-8572 Japan

## Correction to: Journal of Plant Research (2018) 131:961–972 10.1007/s10265-018-1052-7

The article Why is chlorophyll* b* only used in light-harvesting systems?, written by Atsushi Kume, Tomoko Akitsu, Kenio Nishida Nasahara, was originally published electronically on the publisher’s internet portal (currently SpringerLink) on 10 July 2018 without open access.

With the author(s)’ decision to opt for Open Choice the copyright of the article changed on 15 April 2019 to © The Author(s) [2019] and the article is forthwith distributed under the terms of the Creative Commons Attribution 4.0 International License (http://creativecommons.org/licenses/by/4.0/), which permits use, duplication, adaptation, distribution and reproduction in any medium or format, as long as you give appropriate credit to the original author(s) and the source, provide a link to the Creative Commons license and indicate if changes were made.

The original article was corrected.

